# Supplementary figures and images for: Single Cell and Single Nucleus RNA-Seq Reveal Cellular Heterogeneity and Homeostatic Regulatory Networks in Adult Mouse Stria Vascularis
Source: Front Mol Neurosci. 2019 Dec 20;12:316. doi: 10.3389/fnmol.2019.00316 (PMC6933021; doi:10.3389/fnmol.2019.00316)

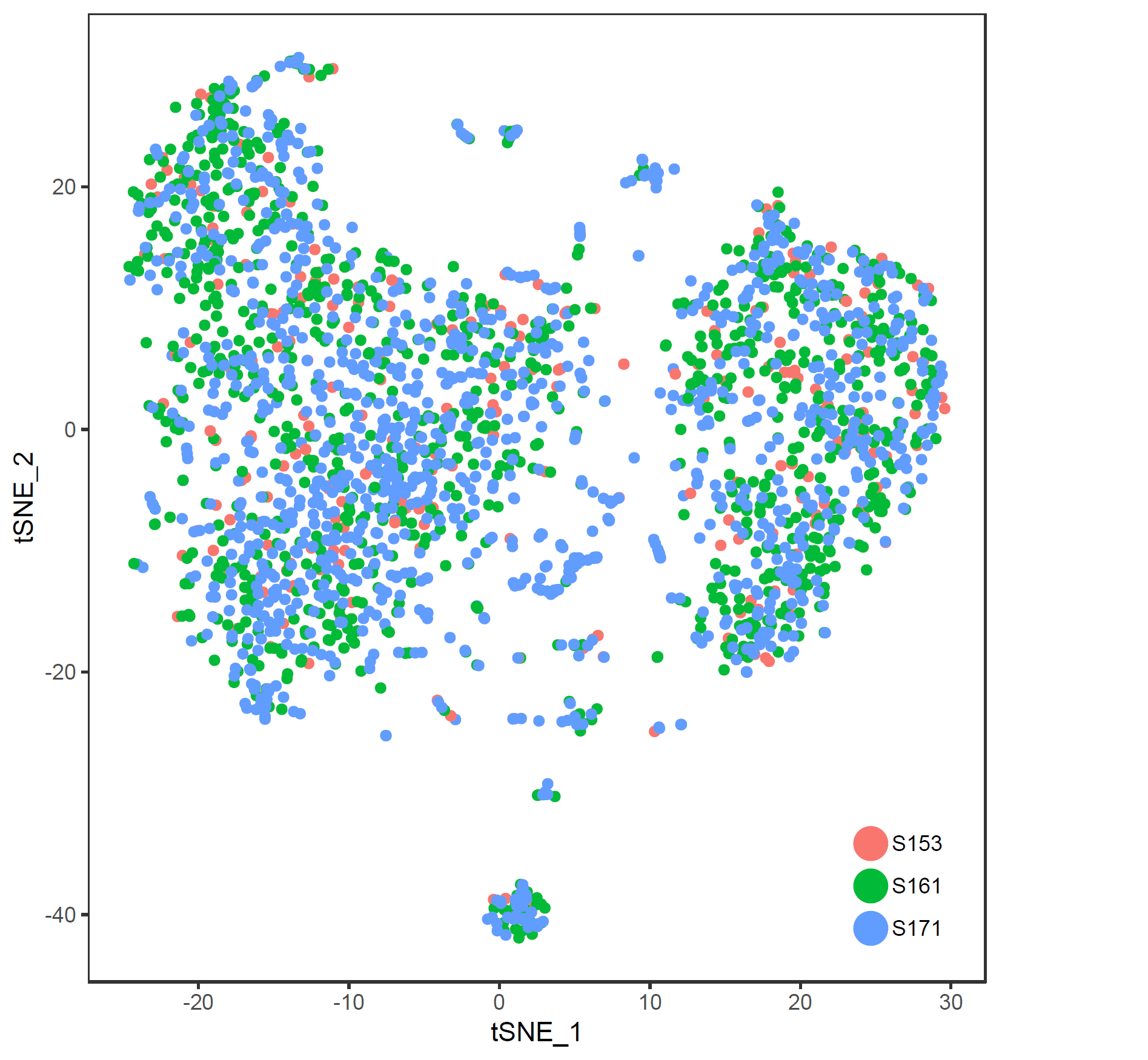

Supplement: FIGURE S1 — Distribution of single cell captures for adult SV scRNA-Seq dataset. Distribution of SV cell captures (S153 in pink, S161 in green, S171 in blue) are equally distributed across all clusters in the scRNA-Seq dataset. S153, S161, and S171 refer to batches collected on separate dates. [file Image_1.TIF]

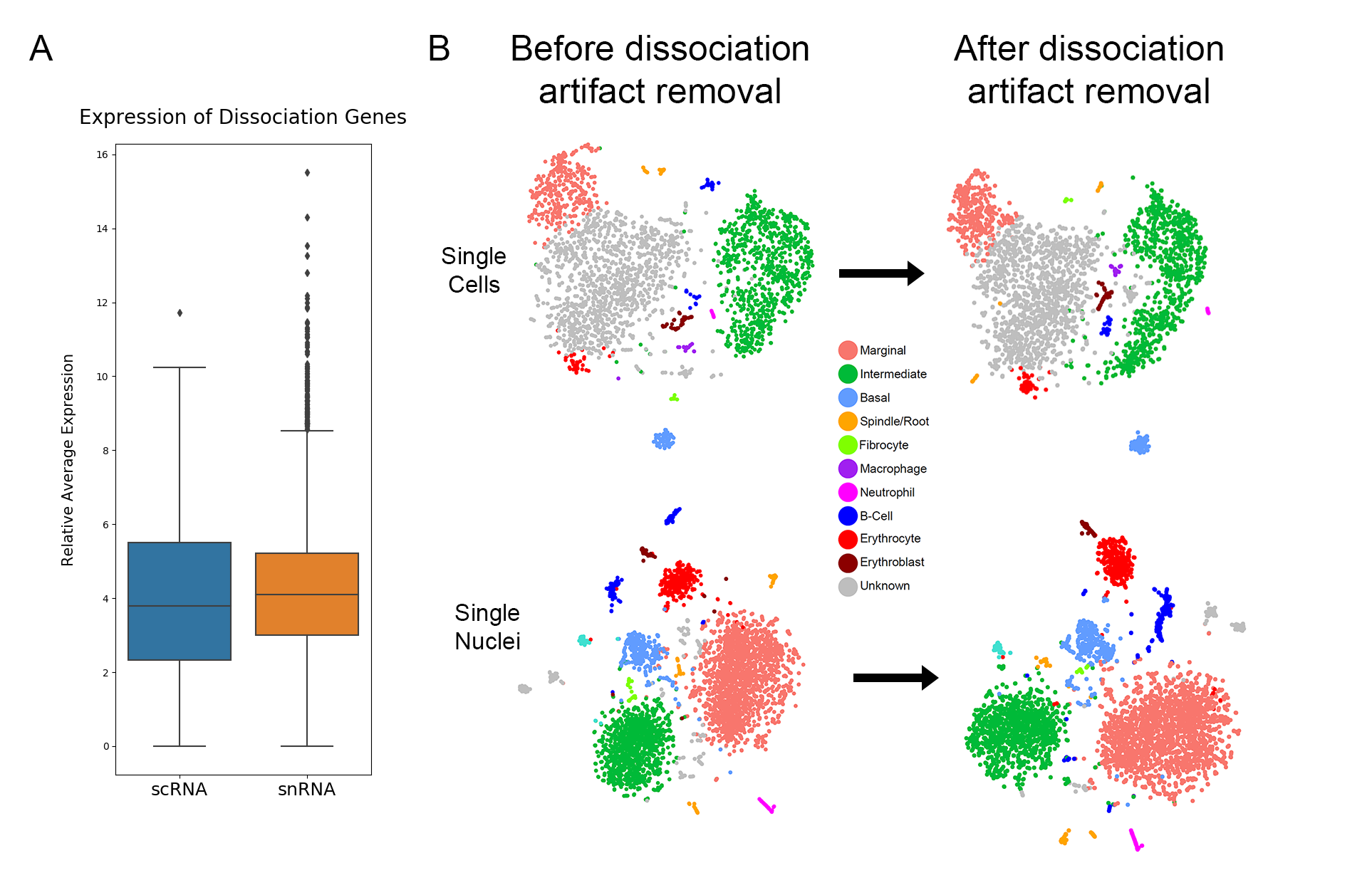

Supplement: FIGURE S2 — Dissociation-induced gene expression in adult mouse SV scRNA-Seq and snRNA-Seq dataset has minimal effect on clustering. (A) Boxplot demonstrating quantification of averaged dissociation-induced gene expression across all cells in the scRNA-Seq dataset (blue box) compared to expression across all nuclei in the snRNA-Seq dataset (orange box). The average dissociation-induced gene expression represents relative level of dissociation-induced gene expression to nuclear loading controls in both single cell and single nucleus datasets. This analysis demonstrates that the level of dissociation-induced gene expression is similar between single cell and single nucleus datasets. See Supplementary Data and Methods for methodology and rationale. Difference in average expression is not statistically significant (p = 0.68). (B) tSNE plots demonstrate clustering of cells and nuclei before and after removal of dissociation artifact and show no difference in the number of clusters. [file Image_2.TIF]

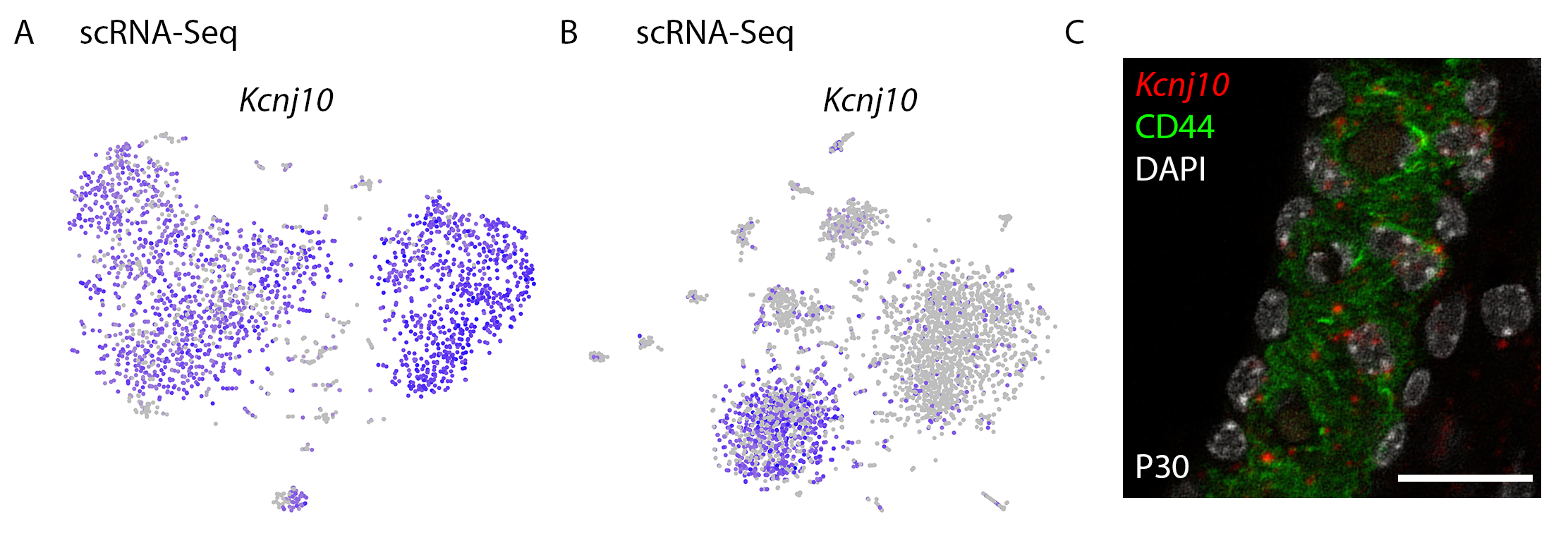

Supplement: FIGURE S3 — snRNA-Seq resolves conflicting results in Kcnj10 expression between scRNA-Seq and snRNA-Seq datasets in the adult mouse stria vascularis. (A) Feature plot from scRNA-Seq dataset demonstrating widespread Kcnj10 expression across cell type clusters. (B) Feature plot from snRNA-Seq dataset demonstrating predominant expression of Kcnj10 in the intermediate cell cluster as demarcated in Figure 2A. (C) smFISH demonstrates Kcnj10 transcripts confined to intermediate cells labeled with anti-CD44 immunostaining. DAPI labels nuclei. Scale bar 20 μm. [file Image_3.TIF]

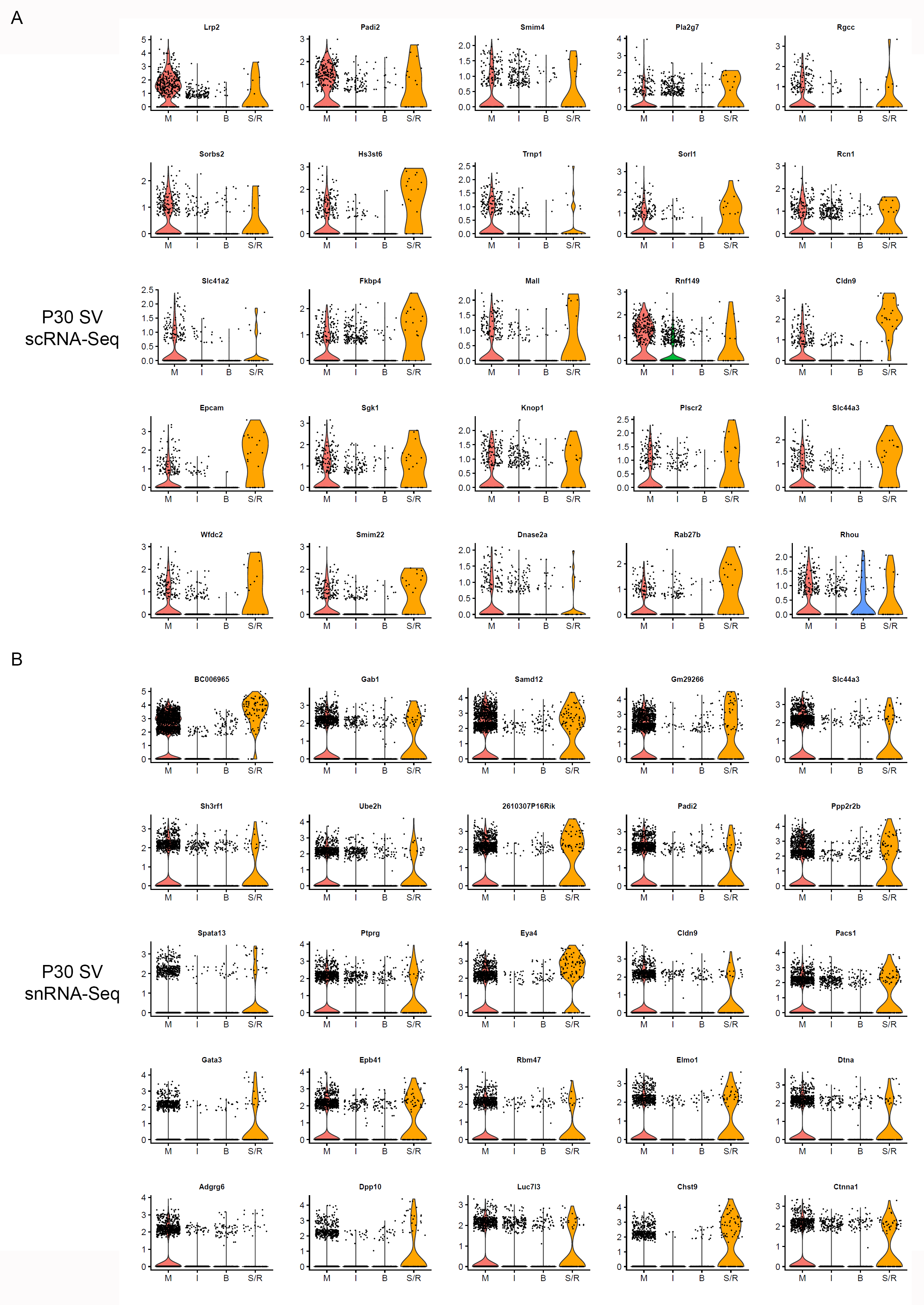

Supplement: FIGURE S4 — Shared gene expression between marginal and spindle cells. (A) Candidate genes identified in the scRNA-Seq dataset expressed by marginal (M) and spindle/root (S/R) cells. (B) Candidate genes identified in the snRNA-Seq dataset expressed by marginal (M) and spindle/root (S/R) cells. Intermediate cells (I) and basal cells (B) are denoted by their respective labels. Violin plots are displayed with normalized counts on the vertical axis and cell types arrayed along the horizontal axis. [file Image_4.TIF]

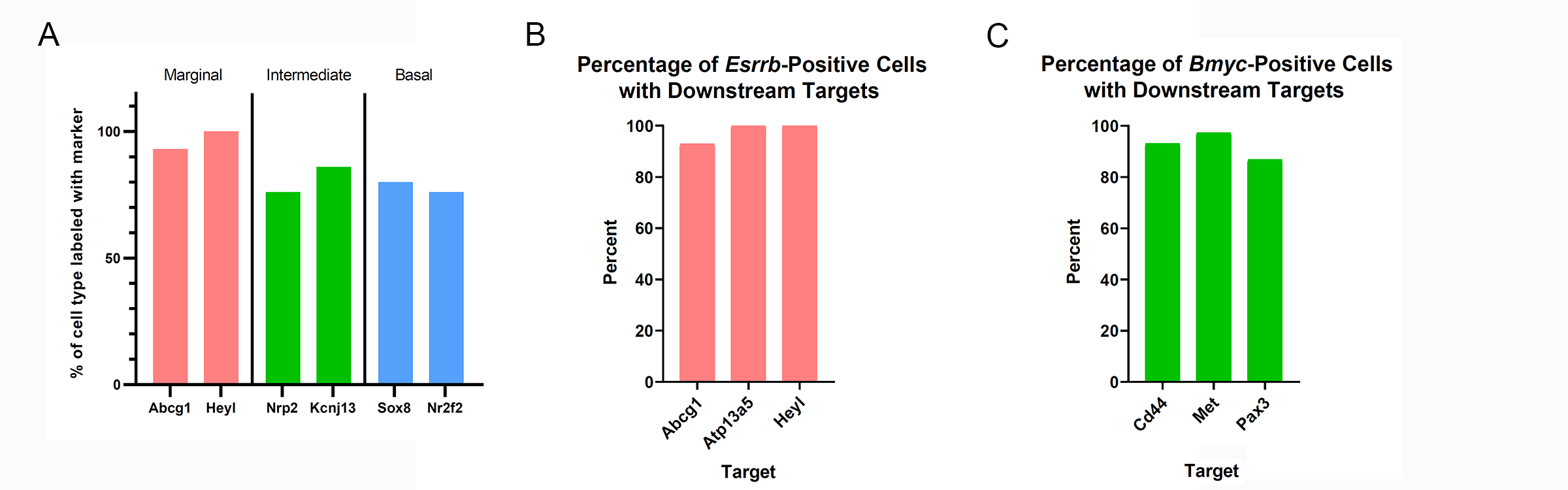

Supplement: FIGURE S5 — smFISH quantification of novel cell type-specific genes and regulon transcription factor with select downstream targets in SV cell types. Customized MATLAB code was utilized to determine the expression of novel gene transcripts in SV cell type nuclei and to determine the number of regulon transcription factor transcript-positive nuclei that expressed each of the downstream gene transcripts. (A) The percentage of cell type-specific nuclei labeled with candidate cell type-specific smFISH probes was quantified. Fifty-two of 56 (93%) and 66 of 66 (100%) of marginal cell nuclei expressed Abcg1 and Heyl transcripts, respectively. One hundred thirty seven of 161 (85%) and 170 of 176 (97%) of Kcnj10- and Cd44-expressing intermediate cell nuclei expressed Nrp2 and Kcnj13 transcripts, respectively. 107 of 145 (73%) and 118 of 185 (64%) of basal cell nuclei express Sox8 (n = 145 cells) and Nr2f2 (n = 185 cells) transcripts, respectively. (B) The percentage of Esrrb transcript-positive nuclei expressing each of the downstream gene transcripts (Abcg1, Atp13a5, Heyl) is shown. Fifty-two of 56 (93%) Esrrb transcript-positive nuclei expressed Abcg1. Fifty-nine of 59 (100%) Esrrb transcript-positive nuclei expressed Atp13a5. Sixty-six of 66 (100%) Esrrb transcript-positive nuclei expressed Heyl. (C) The percentage of Bmyc transcript-positive nuclei expressing each of the downstream gene transcripts (Cd44, Met, Pax3) is shown. Forty of 43 (93%) Bmyc transcript-positive nuclei expressed Cd44. Thirty-six of 37 (97%) Bmyc transcript-positive nuclei expressed Met. Thirty-three of 38 (87%) Bmyc transcript-positive nuclei expressed Pax3. [file Image_5.TIF]

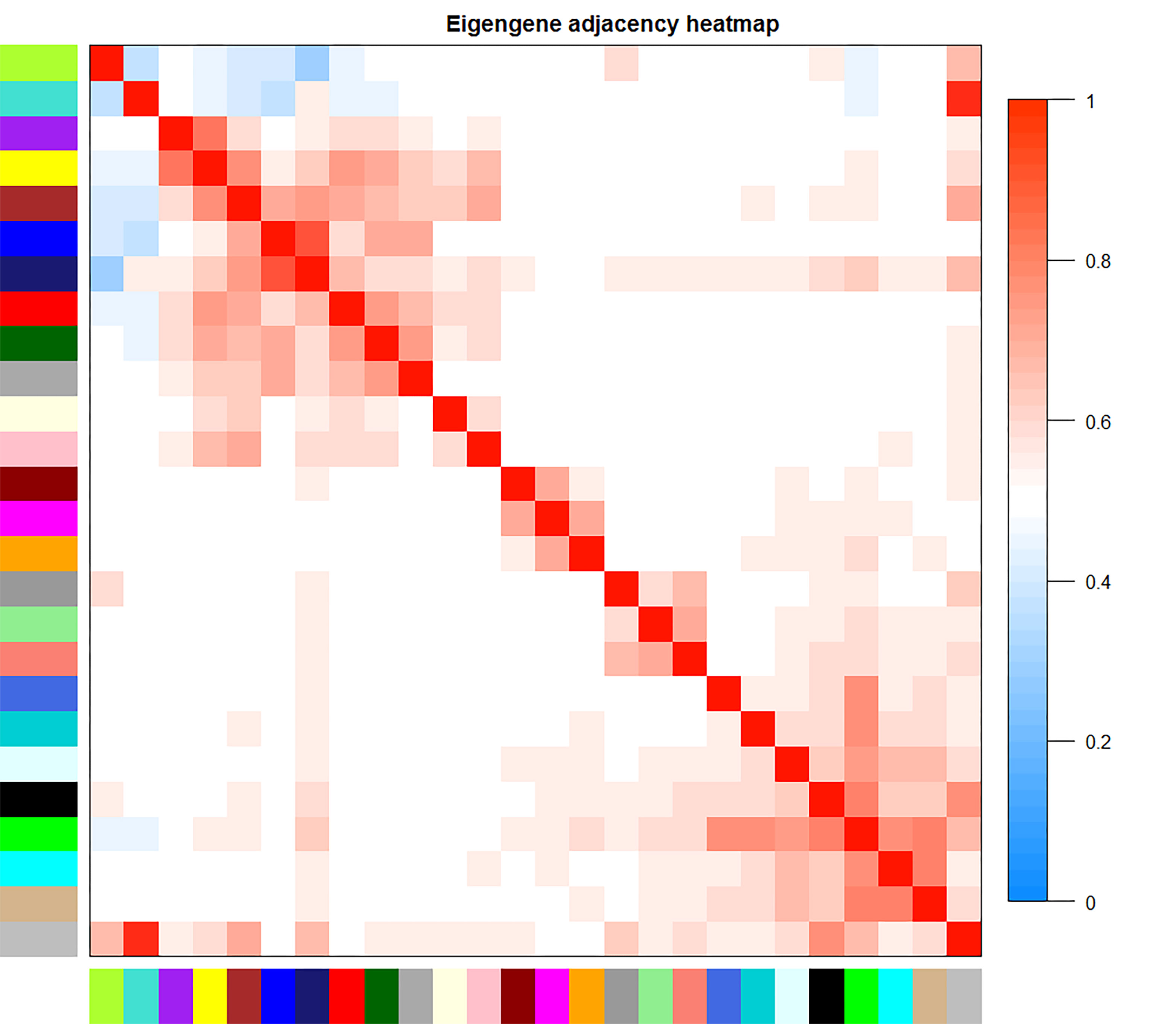

Supplement: FIGURE S6 — Enlarged image of adjacency plot for the scRNA-Seq dataset from Figure 4B. The more red a box is, the more similar the 2 intersecting WGCNA modules are to each other. [file Image_6.JPEG]

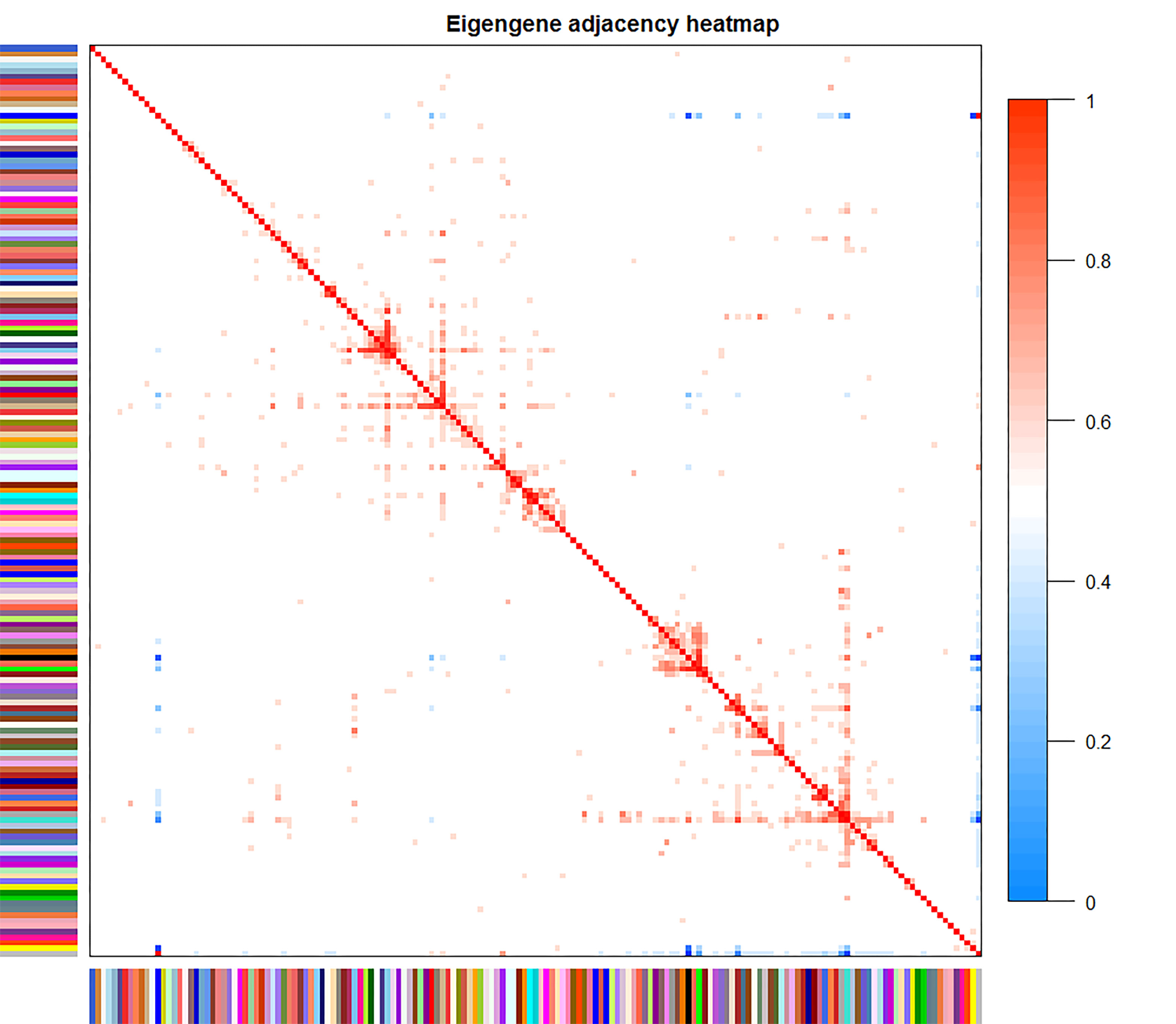

Supplement: FIGURE S7 — Enlarged image of adjacency plot for the snRNA-Seq dataset from Figure 4B. The more red a box is, the more similar the 2 intersecting WGCNA modules are to each other. [file Image_7.JPEG]

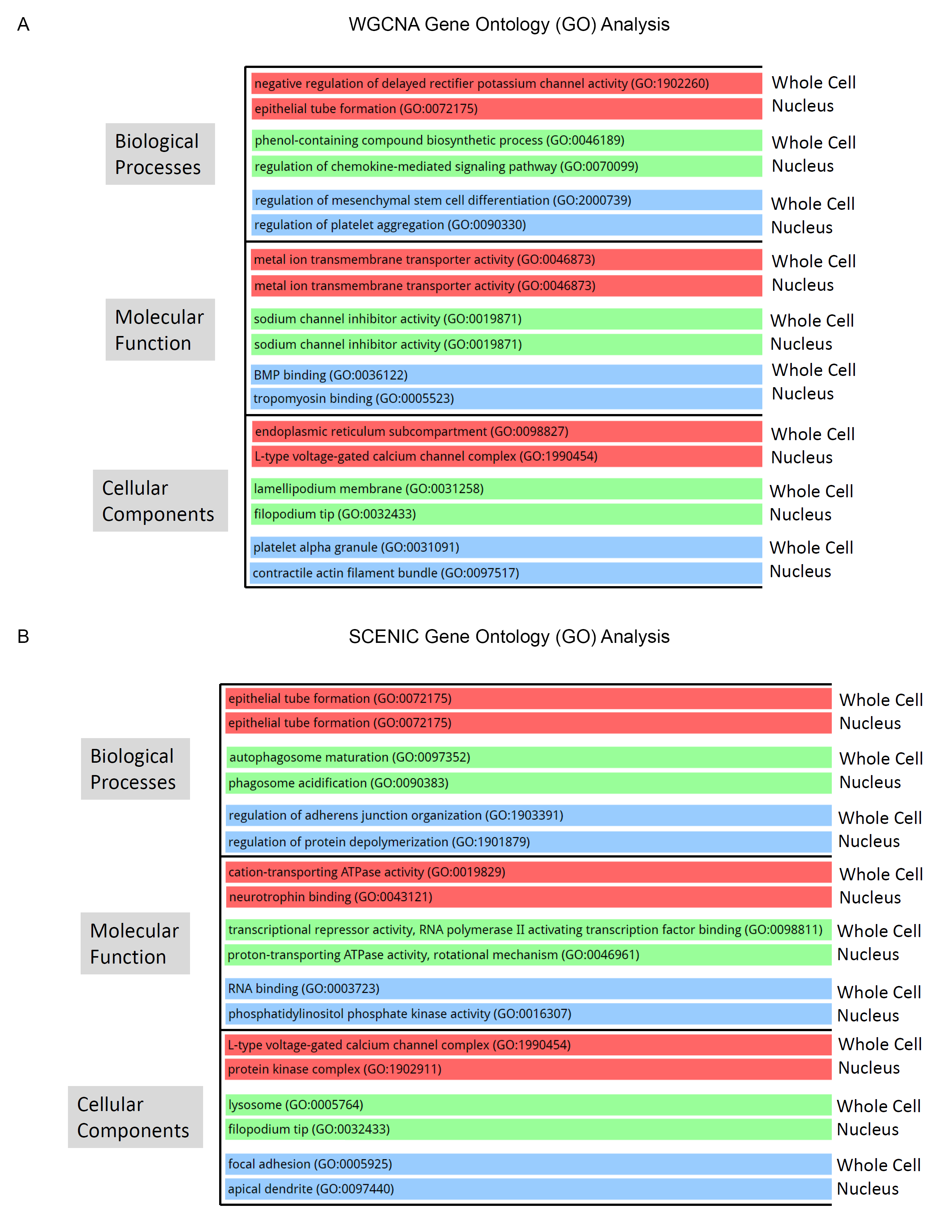

Supplement: FIGURE S8 — Top gene ontology (GO) terms identified by GO analysis with Enrichr in cell type-specific WGCNA modules and cell type-specific SCENIC regulons. (A) GO biological process, molecular function, and cellular component analysis of WGCNA modules from both the scRNA-Seq and snRNA-Seq datasets reveal gene set enrichment in marginal cells (red), intermediate cells (green), and basal cells (blue). (B) GO biological process, molecular function, and cellular component analysis of SCENIC regulons from both the scRNA-Seq and snRNA-Seq datasets reveal gene set enrichment in marginal cells (red), intermediate cells (green), and basal cells (blue). [file Image_8.TIF]

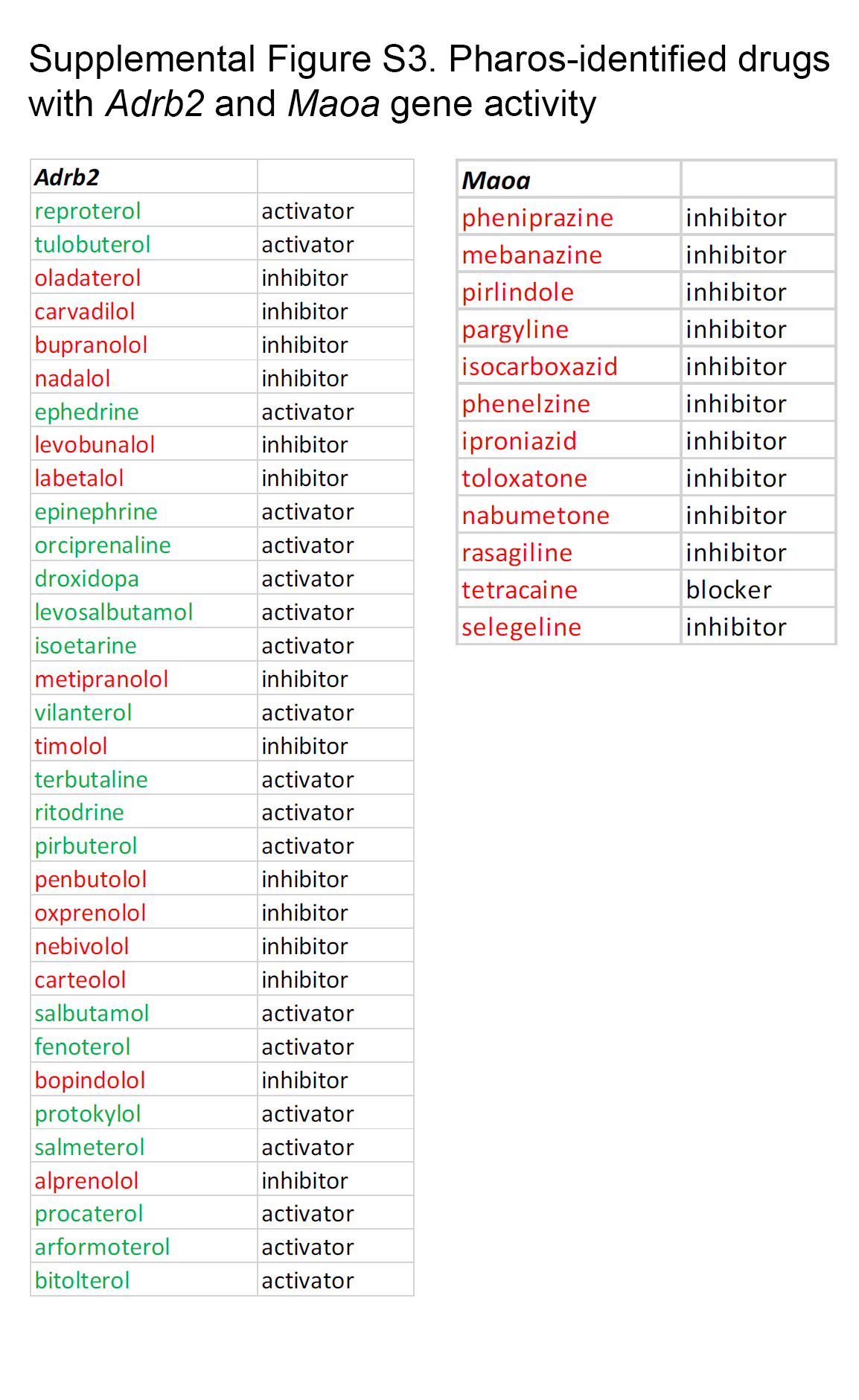

Supplement: FIGURE S9 — Pharos-identified drugs with Adrb2 and Maoa gene activity. Drugs are displayed along with their mechanism of action. Activators are in green and inhibitors are in red. [file Image_9.TIF]
