# Supplementary material for: Single Cell and Single Nucleus RNA-Seq Reveal Cellular Heterogeneity and Homeostatic Regulatory Networks in Adult Mouse Stria Vascularis
Source: Front Mol Neurosci. 2019 Dec 20;12:316. doi: 10.3389/fnmol.2019.00316 (PMC6933021; doi:10.3389/fnmol.2019.00316)
Supplement: TABLE S1 — Key resources. [file Table_1.docx]

**Supplemental Table S1: Key Resources**

| Reagent type  (Species) or Resource | Designation | Source or Reference | Identifiers | Additional Information |
| --- | --- | --- | --- | --- |
| antibody | anti-CD44 (rat monoclonal) | BD Biosciences | BD 550538; AB_393732 | (1:100) |
| antibody | anti-CLDN11 (rabbit polyclonal) | AbCam | ab53041; AB_2276205 | (1:100) |
| antibody | anti-KCNJ10 (rabbit polyclonal) | Alomone labs | APC-035; AB_2040120 | (1:100) |
| antibody | anti-KCNJ16 (rabbit polyclonal) | Sigma Aldrich | SAB4501636; AB_10747844 | (1:100) |
| antibody | anti-KCNQ1 (goat polyclonal) | Sigma Aldrich | SAB2501224; AB_10626617 | (1:100) |
| antibody | anti-SLC12A2 (goat polyclonal) | Santa Cruz | SC-21547; AB_2285988 | (1:100) |
| antibody | anti-ZO-1 (mouse monoclonal) | Fisher Scientific | 339100; AB_2533147 | (1:100) |
| RNAScope probe | *Abcg1* | Advanced Cell Diagnostics | 422221-C2 | targeted region: 3680 – 4595  Accession No: NM_009593.2 |
| RNAScope probe | *Atp13a5* | Advanced Cell Diagnostics | 417211-C1 | targeted region: 2060 – 4007  Accession No: NM_175650.4 |
| RNAScope probe | *Atp1b2* | Advanced Cell Diagnostics | 417131-C1 | targeted region: 968 – 2027  Accession No: NM_013415.5 |
| RNAScope probe | *Bmyc* | Advanced Cell Diagnostics | 593551-C3 | targeted region: 2 – 807  Accession No: NM_023326.2 |
| RNAScope probe | *Cd44* | Advanced Cell Diagnostics | 479191-C1 | targeted region: 2 – 947  Accession No: NM_009851.2 |
| RNAScope probe | *Esrrb* | Advanced Cell Diagnostics | 565951-C3 | targeted region: 437 – 1577  Accession No: NM_001159500.1 |
| RNAScope probe | *Heyl* | Advanced Cell Diagnostics | 446881-C1 | targeted region: 425 – 1345  Accession No: NM_013905.3 |
| RNAScope probe | *Kcne1* | Advanced Cell Diagnostics | 541301-C1 | targeted region: 6 – 929  Accession No: NM_008424.3 |
| RNAScope probe | *Kcnj10* | Advanced Cell Diagnostics | 458831-C3 | targeted region: 498-1441  Accession No: NM_001039484.1 |
| RNAScope probe | *Kcnj13* | Advanced Cell Diagnostics | 412551-C3 | targeted region: 2 – 1098  Accession No: NM_001159500.1 |
| RNAScope probe | *Kcnj16* | Advanced Cell Diagnostics | 492481-C3 | targeted region: 2 – 961  Accession No: NM_001252207.1 |
| RNAScope probe | *Kcnq1* | Advanced Cell Diagnostics | 420481-C2 | targeted region: 6 – 874  Accession No: NM_008434.2 |
| RNAScope probe | *Lrp2* | Advanced Cell Diagnostics | 487751-C1 | targeted region: 1230 – 2350  Accession No: NM_001247984.1 |
| RNAScope probe | *Met* | Advanced Cell Diagnostics | 405301-C2 | targeted region: 3341 – 4309  Accession No: NM_008591.2 |
| RNAScope probe | *Nr2f2* | Advanced Cell Diagnostics | 480301-C1 | targeted region: 1532 – 3193  Accession No: NM_009697.3 |
| RNAScope probe | *Nrp2* | Advanced Cell Diagnostics | 500661-C1 | targeted region: 2001 – 2870  Accession No: NM_001077406.1 |
| RNAScope probe | *P2rx2* | Advanced Cell Diagnostics | 443681-C2 | targeted region: 228 – 1394  Accession No: NM_153400.4 |
| RNAScope probe | *Pax3* | Advanced Cell Diagnostics | 455801-C1 | targeted region: 697 – 1675  Accession No: NM_008781.4 |
| RNAScope probe | *Sox8* | Advanced Cell Diagnostics | 454781-C1 | targeted region: 931 – 1876  Accession No: NM_011447.3 |
| RNAScope probe | *Slc26a4* | Advanced Cell Diagnostics | 452491-C1 | targeted region: 795 – 1776  Accession No: NM_011867.3 |
| embedding medium |  | Section-Lab | SCEM | <http://section-lab.jp/index.html> |
| Cryofilm |  | Section-Lab | type 2C (Adhesive film) | <http://section-lab.jp/index.html> |
| mRNA-Seq assay kit | Chromium Single Cell 3' GEM, Library Gel Bead Kit v3, 16 rxns | 10x Genomics | 1000075 |  |
| mRNA-Seq assay kit | Chromium Chip B Single Cell Kit, 48 rxns | 10x Genomics | 1000073 |  |
| Cell viability assay | LIVE/DEAD Viability/Cytotoxicity Kit | Invitrogen | L3224 |  |
| Cell strainer | 10 μm | pluriSelect Life Science | 45-50010-03 |  |
| Cell strainer | 20 μm | pluriSelect Life Science | 45-50010-03 |  |
| Deposited Data | P30 mouse scRNA-Seq dataset | this paper | GEO Accession ID: GSE136196 |  |
| Deposited Data | P30 mouse snRNA-Seq dataset | this paper | GEO Accession ID: GSE136196 |  |
| strain, strain background (mouse) | CBA/J | The Jackson Laboratory | strain 000656 |  |
| software, algorithm | Seurat v3 |  | RRID:SCR_016341 | https://satijalab.org/seurat/ |
| software, algorithm | WGCNA |  | RRID:SCR_003302 | [https://horvath.genetics.ucla.edu/html/ CoexpressionNetwork/Rpackages/WGCNA/](https://horvath.genetics.ucla.edu/html/%20CoexpressionNetwork/Rpackages/WGCNA/) |
| software, algorithm | SCENIC |  | RRID:SCR_017247 | https://github.com/aertslab/SCENIC |
| software, algorithm | Python |  | 3.6.8; RRID:SCR_008394 | https://www.python.org/ |
| software, algorithm | Pandas |  | 0.24.2; RRID:SCR_000633 | https://pandas.pydata.org/ |
| software, algorithm | Numpy |  | 1.16.4; RRID:SCR_008633 | https://www.numpy.org/ |
| software, algorithm | Matplotlib |  | 3.1.0; RRID:SCR_008624 | https://matplotlib.org/ |
| software, algorithm | Seaborn |  | 0.9.0; | https://seaborn.pydata.org/ |
| software, algorithm | smFISH Quant | this paper |  |  |
