# Supplementary material for: Single Cell and Single Nucleus RNA-Seq Reveal Cellular Heterogeneity and Homeostatic Regulatory Networks in Adult Mouse Stria Vascularis
Source: Front Mol Neurosci. 2019 Dec 20;12:316. doi: 10.3389/fnmol.2019.00316 (PMC6933021; doi:10.3389/fnmol.2019.00316)
Supplement: TABLE S2 — Comparison of single-cell RNA-Seq (scRNA-Seq) and single-nucleus RNA-Seq in the adult stria vascularis. [file Table_2.docx]

**Supplemental Table S2. Comparison of single-cell RNA-Seq (scRNA-Seq) and single-nucleus RNA-Seq in the adult stria vascularis.**

|  | SV scRNA-Seq | SV snRNA-Seq |
| --- | --- | --- |
| Ease of obtaining high numbers of cells or nuclei | Heterogeneity in cell shape and size may affect cells captured | Nuclear heterogeneity less of an issue but small nuclei may represent a separate challenge |
| Affect on cell viability | Single cell preparation harsh on adult fragile tissue | Single nucleus preparation easy on tissue |
| Ease of cell dissociation | Single cell suspension may contain pieces of different cell types stuck together in the case of cell types with interdigitations | Nucleus preparation overcomes issues related to cellular interdigitation or cell types with long processes |
| Optimal uses | May be more optimal for gene regulatory network inference | May achieve better cluster separation of cell types |
