# Supplementary material for: Single Cell and Single Nucleus RNA-Seq Reveal Cellular Heterogeneity and Homeostatic Regulatory Networks in Adult Mouse Stria Vascularis
Source: Front Mol Neurosci. 2019 Dec 20;12:316. doi: 10.3389/fnmol.2019.00316 (PMC6933021; doi:10.3389/fnmol.2019.00316)
Supplement: Supplementary file 18 [file Data_Sheet_1.docx]

**SCENIC to Seurat projection code**

To project SCENIC regulons onto Seurat objects, we loaded the SCENIC expression files and Seurat object and simply substituted the TSNE coordinates of the SCENIC clustering with the TSNE coordinates of the Seurat object. Also included is a function that allows the user to project “extended” regulons in addition to standard regulons.

**# Adjusted function to include extended genes**

**onlyNonDuplicatedExtended <- function (regulonNames){**

**regulonNames <- unname(regulonNames)**

**tfs <- getTF(regulonNames)**

**tfs <- gsub("", "", tfs)**

**splitRegulons <- split(regulonNames, tfs)[unique(tfs)]**

**ret <- sapply(splitRegulons, function(x) {**

**split(x, grepl("", x))[[1]]**

**})**

**return(ret)**

**}**

**# Set working directory to SCENIC results folder**

**setwd("~/SCENIC")**

**# Mount SCENIC package**

**library(SCENIC)**

**# Load SCENIC options and expression matrix**

**load("~/scenicOptions.Rds")**

**load("~/exprMatrix.Rds")**

**# Load the Seurat object to project the SCENIC regulon onto**

**load(“~/SeuratObject”)**

**# Load regulon AUC scores**

**aucell_regulonAUC <- loadInt(scenicOptions, "aucell_regulonAUC")**

**# Substitute Seurat object TSNE coordinates for SCENIC TSNE coordinates**

**AUCell::AUCell_plotTSNE(SeuratObject@dr$tsne@cell.embeddings, exprMatrix,aucell_regulonAUC[onlyNonDuplicatedExtended(rownames(aucell_regulonAUC))["Bmyc_extended"],], plots = "AUC", cex = 0.8, offColor = "grey")**

**WGCNA to Seurat projection code**

To project WGCNA modules onto Seurat objects, we imported the gene list of the WGCNA module of interest, converted them to a character vector, and added them to the “2.6_regulons_asGeneSet” file in the /int folder of the SCENIC file under a new name. This is the file that SCENIC recognizes as the master list of regulon names and gene lists. The “runSCENIC3” function, part of which is the regulon scoring for cells, is re-run so that the new pseudo-regulon of WGCNA module genes will be scored in addition to the SCENIC regulons. We then loaded the SCENIC expression files and Seurat object and substituted the TSNE coordinates of the SCENIC clustering with the TSNE coordinates of the Seurat object, and used the name of the new “WGCNA regulon” as the argument for projection.

**# Import WGCNA module genes**

**TurquoiseGenes <- read.delim("~/TurquoiseGenes.txt", header = FALSE)**

**# Convert gene names into a character vector**

**TurquoiseGenes <- as.character(TurquoiseGenes $V1)**

**# Import 2.6_regulons_asGeneSet file from SCENIC input folder**

**Regulons <- readRDS(file = "~/SCENIC/int/2.6_regulons_asGeneSet.Rds")**

**# Assign WGCNA module gene list to SCENIC regulon list with name of choice**

**Regulons[["ExampleName"]] <- TurquoiseGenes**

**# Mount SCENIC package**

**library(SCENIC)**

**# Save new 2.6_regulons_asGeneSet with added “WGCNA regulon”**

**# It is recommended that this is saved in a new folder so the original SCENIC file is not overwritten**

**saveRDS(Regulons, file = "~/SCENIC/WGCNA/int/2.6_regulons_asGeneSet.Rds")**

**# Set working directory to *new* SCENIC results folder**

**setwd("~/SCENIC/WGCNA")**

**# Load SCENIC options and expression matrix, in this case they are in the new folder to avoid overwriting the original SCENIC data**

**load("~/SCENIC/WGCNA/scenicOptions.Rds")**

**load("~/SCENIC/WGCNA/exprMatrix.Rds")**

**# Load the Seurat object to project the “WGCNA regulon” onto**

**load(“~/SeuratObject”)**

**# Run the SCENIC3 in order to score cells for “WGCNA module”**

**runSCENIC_3_scoreCells(scenicOptions, exprMatrix)**

**# Load regulon AUC scores**

**aucell_regulonAUC <- loadInt(scenicOptions, "aucell_regulonAUC")**

**# Substitute Seurat object TSNE coordinates for SCENIC TSNE coordinates and the regulon name with the “WGCNA regulon” name**

**AUCell::AUCell_plotTSNE(SeuratObject@dr$tsne@cell.embeddings, exprMatrix,aucell_regulonAUC[onlyNonDuplicatedExtended(rownames(aucell_regulonAUC))["ExampleName"],], plots = "AUC", cex = 0.8, offColor = "grey")**
