# Supplementary material for: Single Cell and Single Nucleus RNA-Seq Reveal Cellular Heterogeneity and Homeostatic Regulatory Networks in Adult Mouse Stria Vascularis
Source: Front Mol Neurosci. 2019 Dec 20;12:316. doi: 10.3389/fnmol.2019.00316 (PMC6933021; doi:10.3389/fnmol.2019.00316)
Supplement: Supplementary file 19 [file Data_Sheet_2.docx]

**smFISH Quant: Customized MATLAB code for Quantification of smFISH Transcript Expression**

**cellSpecific function**

function matrix = cellSpecificAuto(im,im2)

% this function allows for quantification of multiple cells designated by

% nuclei. Input is im (image with all channels) and im2 (image without

% DAPI). Output is a matrix with the counts for each cell circled

close all

%import and read image (without dots)

im = imread(im);

figure,imshow(im)

im2 = imread(im2);

original = im;

original2 = im2;

%set variables

choice = 1;

k = 1;

%loop to circle multiple cells

while choice == 1 || choice == 3

close all

figure,imshow(im)

reg = imfreehand(); %select region

M = ~reg.createMask(); % makes everything outside of the selected region take on M

[x,y] = size(M); %takes the matrix of outside the region of interest

L=zeros(x,y,3);

% sets everything outside of the region of interest to 0

for i = 1:x

for j = 1:y

L(i,j,1) = M(i,j);

L(i,j,2) = M(i,j);

L(i,j,3) = M(i,j);

end

end

final = logical(L);

im2(final) = 0;

figure('Name','Cropped')

imshow(im2)

title('Cropped Image')

hold off

mask = reg.createMask();

im(mask) = 0;

%count the number of dots in the region

redimage = im2;

% hold on

% figure('Name','red channel 1')

% title('red channel')

redimage(:,:,2)=0;

redimage(:,:,3)=0;

% imshow(redimage)

% hold off

newR1 = rgb2gray(redimage);

intensityRed = mean(newR1(:));

%count number of red dots

redChannel = redimage(:, :, 1);

redDots = redChannel > 100;

[~, numberOfRedDots] = bwlabel(redDots);

%green channel isolation - planes one and three need to be 0

greenimage = im2;

% hold on

% figure('Name','green channel 1')

% title('green channel')

greenimage(:,:,1)=0;

greenimage(:,:,3)=0;

% imshow(greenimage)

% hold off

newG1 = rgb2gray(greenimage);

intensityGreen = mean(newG1(:));

%count number of green dots

greenChannel = greenimage(:, :, 2);

greenDots = greenChannel > 100; % or whatever

[~, numberOfGreenDots] = bwlabel(greenDots);

%blue channel isolation - planes one and two need to be 0

blueimage = im2;

% hold on

% figure('Name','blue channel')

% title('blue channel 1')

blueimage(:,:,1)=0;

blueimage(:,:,2)=0;

% imshow(blueimage)

% hold off

newB1 = rgb2gray(blueimage);

intensityBlue = mean(newB1(:));

%count number of blue dots

blueChannel = blueimage(:, :, 3);

blueDots = blueChannel > 100; % or whatever

[~, numberOfBlueDots] = bwlabel(blueDots);

% prompt for circling another cell

choice = menu('Would you like to select another cell to examine?', 'Yes','No','Redo Last Cell');

if choice == 1 || choice == 2

% if not redoing the cell then set values to matrix

matrix(k,1) = k;

matrix(k,2) = numberOfRedDots;

matrix(k,3) = numberOfGreenDots;

matrix(k,4) = numberOfBlueDots;

k = k +1;

end

%resets images so another cell can be looked at

im = original;

im2 = original2;

end

end

**Circle Code**

function [Section1, Section2,Section3,Intensity1,Intensity2,Intensity3] = countDotCircle(im,im2,radius)

%This function allows the user to select three circles of equal area with

% input radius. im is the image with all channels and im2 is the image

% without DAPI. The output of the function is the red, green, and blue

% counts in each section and the intensities of each section as well. The

% outputs are all matrices of 3 columns to account for all three colors.

%% choose three sections of equal area circles directed by user input

hold on

title('Click three times on the image to create the circles.')

fig=imshow(im);

[x1,y1]=ginput(1);

x1 = x1 - radius/2;

y1 = y1 - radius/2;

region1 = imellipse(gca,[x1 y1 radius radius]);

[x2,y2]=ginput(1);

x2 = x2 - radius/2;

y2 = y2 - radius/2;

region2 = imellipse(gca,[x2 y2 radius radius]);

[x3,y3]=ginput(1);

x3 = x3 - radius/2;

y3 = y3 - radius/2;

region3 = imellipse(gca,[x3 y3 radius radius]);

hold off

%% crop the image into sections

%split into first section image - sets everything outside of the region of

%interest to 0 creating a mask

% hold on

dummy1 = double(region1.createMask());

M1 = ~dummy1;

image1 = imread(im2);

I1 = image1;

[row1,col1] = size(M1);

L1 = zeros(row1,col1,3);

for i = 1:row1

for j = 1:col1

L1(i,j,1) = M1(i,j);

L1(i,j,2) = M1(i,j);

L1(i,j,3) = M1(i,j);

end

end

final1 = logical(L1);

I1(final1) = 0;

% figure ('Name', 'Cropped Region 1')

% imshow(I1)

% title('Cropped Region 1')

% hold off

%split into second section image - sets everything outside of the region of

%interest to 0 creating a mask

% hold on

dummy2 = double(region2.createMask());

M2 = ~dummy2;

image2 = imread(im2);

I2 = image2;

[row2,col2] = size(M2);

L = zeros(row2,col2,3);

for i = 1:row2

for j = 1:col2

L2(i,j,1) = M2(i,j);

L2(i,j,2) = M2(i,j);

L2(i,j,3) = M2(i,j);

end

end

final2 = logical(L2);

I2(final2) = 0;

% figure ('Name', 'Cropped Region 2')

% imshow(I2)

% title('Cropped Region 2')

% hold off

%split into third section image - sets everything outside of the region of

%interest to 0 creating a mask

% hold on

dummy3 = double(region3.createMask());

M3 = ~dummy3;

image3 = imread(im2);

I3 = image3;

[row3,col3] = size(M3);

L3 = zeros(row3,col3,3);

for i = 1:row3

for j = 1:col3

L3(i,j,1) = M3(i,j);

L3(i,j,2) = M3(i,j);

L3(i,j,3) = M3(i,j);

end

end

final3 = logical(L3);

I3(final3) = 0;

% figure ('Name', 'Cropped Region 3')

% imshow(I3)

% title('Cropped Region 3')

% hold off

%% for cropped region R - addressing the first circle

%red channel isolation - planes two and three need to be 0

redimage = I1;

% hold on

% figure('Name','red channel 1')

% title('red channel')

redimage(:,:,2)=0;

redimage(:,:,3)=0;

% imshow(redimage)

% hold off

newR1 = rgb2gray(redimage);

intensityRed(1) = mean(newR1(:)); % takes intensities

%count number of red dots

redChannel = redimage(:, :, 1);

redDots = redChannel > 100;

[~, numberOfRedDots(1)] = bwlabel(redDots);

% fprintf('Number of Red Dots in Section 1: %d\n', numberOfRedDots(1))

%green channel isolation - planes one and three need to be 0

greenimage = I1;

% hold on

% figure('Name','green channel 1')

% title('green channel')

greenimage(:,:,1)=0;

greenimage(:,:,3)=0;

% imshow(greenimage)

% hold off

newG1 = rgb2gray(greenimage);

intensityGreen(1) = mean(newG1(:)); % takes intensity

%count number of green dots

greenChannel = greenimage(:, :, 2);

greenDots = greenChannel > 100; % or whatever

[~, numberOfGreenDots(1)] = bwlabel(greenDots);

% fprintf('Number of Green Dots in Section 1: %d\n', numberOfGreenDots(1))

%blue channel isolation - planes one and two need to be 0

blueimage = I1;

% hold on

% figure('Name','blue channel')

% title('blue channel 1')

blueimage(:,:,1)=0;

blueimage(:,:,2)=0;

% imshow(blueimage)

% hold

newB1 = rgb2gray(blueimage);

intensityBlue(1) = mean(newB1(:)); % takes intensity

%count number of blue dots

blueChannel = blueimage(:, :, 3);

blueDots = blueChannel > 100; % or whatever

[~, numberOfBlueDots(1)] = bwlabel(blueDots);

% fprintf('Number of Blue Dots in Section 1: %d\n', numberOfBlueDots(1))

%% for cropped region S - addresses the second circle

%red channel isolation - planes two and three need to be 0

redimage = I2;

% hold on

% figure('Name','red channel')

% title('red channel 2')

redimage(:,:,2)=0;

redimage(:,:,3)=0;

% imshow(redimage)

% hold off

newR2 = rgb2gray(redimage);

intensityRed(2) = mean(newR2(:));

%count number of red dots

redChannel = redimage(:, :, 1);

redDots = redChannel > 100;

[~, numberOfRedDots(2)] = bwlabel(redDots);

% fprintf('Number of Red Dots in Section 2: %d\n', numberOfRedDots(2))

%green channel isolation - planes one and three need to be 0

greenimage = I2;

% hold on

% figure('Name','green channel')

% title('green channel 2')

greenimage(:,:,1)=0;

greenimage(:,:,3)=0;

% imshow(greenimage)

% hold off

newG2 = rgb2gray(greenimage);

intensityGreen(2) = mean(newG2(:));

%count number of green dots

greenChannel = greenimage(:, :, 2);

greenDots = greenChannel > 100; % or whatever

[~, numberOfGreenDots(2)] = bwlabel(greenDots);

% fprintf('Number of Green Dots in Section 2: %d\n', numberOfGreenDots(2))

%blue channel isolation - planes one and two need to be 0

blueimage = I2;

% hold on

% figure('Name','blue channel')

% title('blue channel 2')

blueimage(:,:,1)=0;

blueimage(:,:,2)=0;

% imshow(blueimage)

% hold off

newB2 = rgb2gray(blueimage);

intensityBlue(2) = mean(newB2(:));

%count number of blue dots

blueChannel = blueimage(:, :, 3);

blueDots = blueChannel > 100; % or whatever

[~, numberOfBlueDots(2)] = bwlabel(blueDots);

% fprintf('Number of Blue Dots in Section 2: %d\n', numberOfBlueDots(2))

%% for cropped region T - addresses the third circle

%red channel isolation - planes two and three need to be 0

redimage = I3;

% hold on

% figure('Name','red channel')

% title('red channel 3')

redimage(:,:,2)=0;

redimage(:,:,3)=0;

% imshow(redimage)

% hold off

newR3 = rgb2gray(redimage);

intensityRed(3) = mean(newR3(:));

%count number of red dots

redChannel = redimage(:, :, 1);

redDots = redChannel > 100;

[~, numberOfRedDots(3)] = bwlabel(redDots);

% fprintf('Number of Red Dots in Section 3: %d\n', numberOfRedDots(3))

%green channel isolation - planes one and three need to be 0

greenimage = I3;

% hold on

% figure('Name','green channel')

% title('green channel 3')

greenimage(:,:,1)=0;

greenimage(:,:,3)=0;

% imshow(greenimage)

% hold off

newG3 = rgb2gray(greenimage);

intensityGreen(3) = mean(newG3(:));

%count number of green dots

greenChannel = greenimage(:, :, 2);

greenDots = greenChannel > 100; % or whatever

[~, numberOfGreenDots(3)] = bwlabel(greenDots);

% fprintf('Number of Green Dots in Section 3: %d\n', numberOfGreenDots(3))

%blue channel isolation - planes one and two need to be 0

blueimage = I3;

% hold on

% figure('Name','blue channel')

% title('blue channel 3')

blueimage(:,:,1)=0;

blueimage(:,:,2)=0;

% imshow(blueimage)

% hold off

newB3 = rgb2gray(blueimage);

intensityBlue(3) = mean(newB3(:));

%count number of blue dots

blueChannel = blueimage(:, :, 3);

blueDots = blueChannel > 100; % or whatever

[~, numberOfBlueDots(3)] = bwlabel(blueDots);

% fprintf('Number of Blue Dots in Section 3: %d\n', numberOfBlueDots(3))

%% printing and setting values for matrices

%create matrices for dot counts

Section1=[];

Section2=[];

Section3=[];

%set values for dot counts

Section1(1,1) = numberOfRedDots(1);

Section1(1,2) = numberOfGreenDots(1);

Section1(1,3) = numberOfBlueDots(1);

Section2(1,1) = numberOfRedDots(2);

Section2(1,2) = numberOfGreenDots(2);

Section2(1,3) = numberOfBlueDots(2);

Section3(1,1) = numberOfRedDots(3);

Section3(1,2) = numberOfGreenDots(3);

Section3(1,3) = numberOfBlueDots(3);

%create intensity matrices

Intensity1 = [];

Intensity2 = [];

Intensity3 = [];

%set the intensity for approximation of individual dots by dividing total

%intensity of the circle by the number of dots in the circle

Intensity1(1,1) = (intensityRed(1)/numberOfRedDots(1));

Intensity1(1,2) = (intensityGreen(1)/numberOfGreenDots(1));

Intensity2(1,1) = (intensityRed(2)/ numberOfRedDots(2));

Intensity2(1,2) = (intensityGreen(2)/numberOfGreenDots(2));

Intensity3(1,1) = (intensityRed(3)/numberOfRedDots(3));

Intensity3(1,2) = (intensityGreen(3)/numberOfGreenDots(3));

%need to account for number of blue dots being zero, then the intensity of

%the blue ones would just be 0

if numberOfBlueDots(1) == 0

Intensity1(1,3) = 0;

else

Intensity1(1,3) = (intensityBlue(1)/numberOfBlueDots(1));

end

if numberOfBlueDots(2) == 0

Intensity2(1,3) = 0;

else

Intensity2(1,3) = (intensityBlue(2)/numberOfBlueDots(2));

end

if numberOfBlueDots(3) == 0

Intensity3(1,3) = 0;

else

Intensity3(1,3) = (intensityBlue(3)/numberOfBlueDots(3));

end

end

**Freehand Code**

function [numberOfRedDots, numberOfGreenDots, numberOfBlueDots, intensityRed, intensityGreen, intensityBlue] = countDotFree(im,im2)

% This function allows the user to circle any region on the image. Input is

% im (image with all channels) and im2 (image without DAPI). Output is the

% number of dots in the region for each color and the intensities

%reads images into workspace and displays images with all channels

im = imread(im);

figure,imshow(im)

im2 = imread(im2);

close all

figure,imshow(im)

reg = imfreehand(); %select region

M = ~reg.createMask(); % makes everything outside of the selected region take on M

[x,y] = size(M); %takes the matrix of outside the region of interest

L=zeros( x,y,3);

% sets everything outside the region of interest to 0 (black)

for i = 1:x

for j = 1:y

L(i,j,1) = M(i,j);

L(i,j,2) = M(i,j);

L(i,j,3) = M(i,j);

end

end

final = logical(L);

im2(final) = 0;

figure('Name','Cropped')

imshow(im2)

title('Cropped Image')

hold off

mask = reg.createMask();

im(mask) = 0;

%% counts and intensities

%red channel isolation - planes two and three need to be 0

redimage = im2;

% hold on

% figure('Name','red channel 1')

% title('red channel')

redimage(:,:,2)=0;

redimage(:,:,3)=0;

% imshow(redimage)

% hold off

newR1 = rgb2gray(redimage);

intensityRed = mean(newR1(:));

%count number of red dots

redChannel = redimage(:, :, 1);

redDots = redChannel > 100;

[~, numberOfRedDots] = bwlabel(redDots);

% fprintf('Number of Red Dots in Section 1: %d\n', numberOfRedDots(1))

%green channel isolation - planes one and three need to be 0

greenimage = im2;

% hold on

% figure('Name','green channel 1')

% title('green channel')

greenimage(:,:,1)=0;

greenimage(:,:,3)=0;

% imshow(greenimage)

% hold off

newG1 = rgb2gray(greenimage);

intensityGreen = mean(newG1(:));

%count number of green dots

greenChannel = greenimage(:, :, 2);

greenDots = greenChannel > 100; % or whatever

[~, numberOfGreenDots] = bwlabel(greenDots);

% fprintf('Number of Green Dots in Section 1: %d\n', numberOfGreenDots(1))

%blue channel isolation - planes one and two need to be 0

blueimage = im2;

% hold on

% figure('Name','blue channel')

% title('blue channel 1')

blueimage(:,:,1)=0;

blueimage(:,:,2)=0;

% imshow(blueimage)

% hold

newB1 = rgb2gray(blueimage);

intensityBlue = mean(newB1(:));

%count number of blue dots

blueChannel = blueimage(:, :, 3);

blueDots = blueChannel > 100; % or whatever

[~, numberOfBlueDots] = bwlabel(blueDots);

% fprintf('Number of Blue Dots in Section 1: %d\n', numberOfBlueDots(1))

%% printing and setting values for matrices

if numberOfRedDots == 0

intensityRed = 0;

else

intensityRed = (intensityRed/numberOfRedDots);

end

if numberOfGreenDots == 0

intensityGreen = 0;

else

intensityGreen = (intensityGreen/numberOfGreenDots);

end

if numberOfBlueDots == 0

intensityBlue = 0;

else

intensityBlue = (intensityBlue/ numberOfBlueDots);

end

end

**GUI CODE**

function CellSpecificQuantButtonPushed(app, event)

% set variable for sheet to export data to

sheet = 1;

% prompt for what spreadsheet data should be exported to

prompt = 'What is the name of the spreadsheet you would like to export the data to?';

dlgtitle = 'Input';

reply2 = inputdlg(prompt,dlgtitle)

reply2 = char(reply2)

% loop to examine multiple images

while sheet == 1 || strcmp(reply,'Yes') %need to have some sort of condition to end circling of the regions

im = uigetfile('*.tif' ); %image with all channels

im2 = uigetfile('*.tif'); %image without DAPI

% call function to count

matrix = cellSpecificAuto(im,im2);

% put data into spreadsheet

title = ["Cell Number", "Red", "Green", "Blue"];

final = [title; matrix];

xlswrite(reply2,final,sheet)

% prompt for examining another image, sheet number changed for next data entry

reply = questdlg('Would you like to examine another image?');

sheet = sheet + 1;

close all

end

end

% Button pushed function: NonCellSpecificQuantCirclesButton

function NonCellSpecificQuantCirclesButtonPushed(app, event)

% these variables are used for counting the number of cycle (images) that are done and creating the matrices that will be exported

k = 1; dum = 1;M =[];N=[];

% data export location

prompt = 'What is the name of the spreadsheet you would like to export the data to? (including extension name)';

dlgtitle='Input';

reply = inputdlg(prompt,dlgtitle)

reply = char(reply)

% gives options for the radius of the circle

choice = menu('What would you like the radius of your circle to be in pixels?', '100','150','200','250','125');

if choice == 1

radius = 100;

elseif choice == 2

radius = 150;

elseif choice == 3

radius = 200;

elseif choice == 4

radius = 250;

elseif choice == 5

radius = 125;

end

% loop in order to examine multiple images at once

while k == 1 || strcmp(answer,'Yes')

clc % clears area

%user imports the images that will be examined

im = uigetfile('*.tif'); % image with all channels

im2 = uigetfile('*.tif'); % image without DAPI

% calls function to collect data

[Section1, Section2, Section3,Intensity1,Intensity2,Intensity3] = countDotCircle(im,im2,radius);

% set the values of the matrix for output to the values that were collected by the function

% M represents dot counts

M(dum,1:3) = Section1(1:3);

M(dum+1,1:3)= Section2(1:3);

M(dum+2,1:3)= Section3(1:3);

%N represents intensity counts

N(dum,1:3) = Intensity1(1:3);

N(dum+1,1:3)= Intensity2(1:3);

N(dum+2,1:3)= Intensity3(1:3);

% next matrices will print 4 lines later

dum = dum +4;

% prompt for examining another image

k= k+1;

answer = questdlg('Would you like to examine another image?');

if strcmp(answer,'Yes')

close all

end

end

%condenses all matrices and exports to spreadsheet

disp(M)

disp(N)

color = ["Red Count","Green Count","Blue Count","Red Intensity","Green Intensity","Blue Intensity"];

A = [color;M N]

xlswrite(reply,A)

close all

end

% Button pushed function: NonCellSpecificQuantFreehandButton

function NonCellSpecificQuantFreehandButtonPushed(app, event)

% set variables for matrices and counts for the number of images

k = 1; dum = 1; M = []; N = [];

% name of spreadsheet to export to

prompt = 'What is the name of the spreadsheet you would like to export the data to? (including extension name)';

dlgtitle = 'Input';

reply = inputdlg(prompt,dlgtitle)

reply = char(reply)

% loop to examine multiple images

while k ==1 || strcmp(answer,'Yes')

close all, clc %clear workspace

im = uigetfile('*.tif'); %import images with all channels

im2 = uigetfile('*.tif'); % import image without DAPI

% call function to count and find intensities

[numberOfRedDots, numberOfGreenDots, numberOfBlueDots, intensityRed, intensityGreen, intensityBlue] = countDotFree(im,im2)

% set values of final matrix to be exported to spreadsheet

M(dum,1) = numberOfRedDots;

M(dum,2) = numberOfGreenDots;

M(dum,3) = numberOfBlueDots;

N(dum,1) = intensityRed;

N(dum,2) = intensityGreen;

N(dum,3) = intensityBlue;

% prompts and count for examining another image

dum = dum+1;

k = k+1;

answer = questdlg('Would you like to examine another image?');

if strcmp(answer,'Yes')

close all

end

end

% sets value for final matrix for export to spreadsheet

color = ["Red Count", "Green Count", "Blue Count", "Red Intensity", "Green Intensity", "Blue Intensity"];

A = [color; M N]

xlswrite(reply,A)

close all

end
