# Supplementary material for: Single Cell and Single Nucleus RNA-Seq Reveal Cellular Heterogeneity and Homeostatic Regulatory Networks in Adult Mouse Stria Vascularis
Source: Front Mol Neurosci. 2019 Dec 20;12:316. doi: 10.3389/fnmol.2019.00316 (PMC6933021; doi:10.3389/fnmol.2019.00316)
Supplement: DATA AND METHODS — Comparative advantages between scRNA-seq and snRNA-seq in the adult stria vascularis. [file Data_Sheet_3.docx]

**Supplemental Data and Methods**

**Comparative advantages between scRNA-seq and snRNA-seq in the adult stria vascularis.**

Single-cell and single-nucleus RNA-Seq are potentially complementary approaches to understanding the cellular transcriptomes of the stria vascularis. Table 2 highlights the differences in the two techniques as they relate to the SV (Supplemental Table S2).

Isolation of nuclei in the case of the SV which exhibits a fair amount of cellular size heterogeneity, enabled capture of larger numbers of nuclei (versus cells) on most captures. While this may relate to increased familiarity with the technique over time, the use of nuclei isolation for single-nucleus RNA-sequencing has been used for this exact purpose by others (Lacar et al., 2016; Lake et al., 2016; Sathyamurthy et al., 2018; Wu, Kirita, Donnelly, & Humphreys, 2019a; Zeng et al., 2016). As has been previously noted, dissociation of cells may effect viability as well as gene expression (Van Den Brink et al., 2017). For these reasons, both scRNA-Seq and snRNA-Seq protocols have attempted to minimize the time from tissue dissociation to cell capture. For comparison, we used 3 frequently utilized nuclear housekeeping genes (*Lmnb1*, *Pcna*, *Tbp*) (Akiyama et al., 2013; Iwai et al., 2019; Prior et al., 2014; Zhao et al., 2019), as both datasets had material from the nucleus on the basis of isolation methods, to create a composite normalization factor. This normalization factor was utilized to create a mean estimate of total cellular content that would be less sensitive to the technical or biological fluctuations of a single loading control (Janes, 2015). The expression of dissociation-induced genes in each cell underwent normalization and then average expression of dissociation-induced genes was calculated in both the scRNA-Seq and snRNA-Seq datasets. Based on this analysis, the average dissociation-induced gene expression between the scRNA-Seq and snRNA datasets exhibited a difference in the dissociation-induced gene expression average that was not statistically significant (p = 0.68) (Supplemental Figure S2A). More importantly, re-running the clustering of our datasets after removing known dissociation artifact genes (Baryawno et al., 2019; Van Den Brink et al., 2017) did not reveal substantial changes in clustering (Supplemental Figure S2B). Specifically, the numbers of clusters remained the same and greater than 95% of the cells had the same cluster assignment. Regarding the ease of cell dissociation, we noted the presence of seemingly more widespread expression of *Kcnj10* RNA expression in SV cells in the scRNA-Seq dataset (Supplemental Figure S3A) compared to the expected expression pattern isolated to SV intermediate cells in the snRNA-Seq dataset (Supplemental Figure S3B) that was consistent with published literature (Wangemann et al., 2004). Colocalization of *Kcnj10* RNA transcript expression with CD44 protein is consistent with published literature (Ando & Takeuchi, 1999) and snRNA-Seq dataset which confirm *Kcnj10* RNA expression isolated to intermediate cells in the SV (Supplemental Figure S3C). While, the expression of *Kcnj10* is not completely isolated to the intermediate cell cluster (as identified in green in Figure 2) in the snRNA-Seq dataset, it is largely confined to the intermediate cell cluster and expression in other clusters may be related to technical noise or true stochastic expression. Intermediate cells, similar to other cells of melanocytic origin, have been shown to be capable of donating melanosomes to adjacent cells (Cable & Steel, 1991). This suggests that donation of *Kcnj10* RNA-containing vesicles by intermediate cells to marginal cells might be possible. However, Cable and Steel go on to show that while melanosome donation does occur, that the accepting cells are not capable of melanin biosynthesis, suggesting that even if donation occurs, that RNA and protein production themselves are less likely (Cable & Steel, 1991). Furthermore, the failure to localize *Kcnj10* RNA to marginal cells argues against this possibility (Supplemental Figure S3C).

Another possibility for the detection of *Kcnj10* in marginal cells is that detection of ambient RNA is responsible for the detection of *Kcnj10* in the scRNA-Seq dataset in cells outside the intermediate cell cluster and that the snRNA-Seq dataset is less susceptible to the effects of ambient RNA detection. Yang and colleagues have suggested the possibility of cross-contamination by ambient RNA, defined as the mRNA pool released in the cell suspension, likely from stressed or apoptotic cells (Yang et al., 2019). Specifically, recall that intermediate and marginal cells have interdigitating processes and the isolation of nuclei may be less susceptible to the detection of RNA from these interdigitating processes that adhere to other cell types (i.e. intermediate cell process on marginal cell). Alternatively, despite attempting to computationally remove transcriptome profiles derived from doublets or two cells captured in one droplet, it is possible that some of this expression in other clusters is due to transcriptome profiles derived from doublets. Nonetheless, this data suggests that snRNA-Seq may have an advantage over scRNA-Seq when it comes to cell cluster separation.

In contrast, gene regulatory network inference may be more robust in situations where there is a greater number of genes detected in a given cell or nuclei. While some have noted that genes detected per nuclei tend to be lower than genes detected per cell (Bakken et al., 2018), some recent work has suggested the possibility of detecting relatively equivalent numbers of genes per nuclei compared to cells (Wu, Kirita, Donnelly, & Humphreys, 2019b). Our adult SV single-cell and single-nucleus RNA-Seq datasets were generally consistent with published comparisons between scRNA-Seq and snRNA-Seq datasets (Bakken et al., 2018; Habib et al., 2016; Hu et al., 2018; Lacar et al., 2016). The discrepancies in the number of genes detected amongst cell type-specific WGCNA modules between single cell and single nucleus datasets may represent a combination of ambient RNA detection (Yang et al., 2019) amongst intermediate cells mentioned previously and a technical aspect of WGCNA analysis where genes can only be assigned to one module when in reality a given gene may play different roles in different gene regulatory networks (Langfelder & Horvath, 2008). Nonetheless, our analyses in SCENIC as well as WGCNA suggest that the larger number of genes detected per cells versus nuclei may be related to the ability to infer gene regulatory networks. Consequently, the identification of druggable gene targets utilizing Pharos resulted in more Tclin druggable gene targets, which can be targeted by FDA-approved drugs, from the scRNA-Seq versus snRNA-Seq datasets (83 versus 22 genes). With respect to the identification of druggable genes, Pharos identified 88 Tclin druggable gene targets from cell type-specific SCENIC regulons versus 35 Tclin druggable gene targets from cell type-specific WGCNA modules. The difference between the two analyses may be related to the difference in algorithm optimization for analysis of single-cell RNA-sequencing datasets as SCENIC was designed for these datasets while WGCNA was not. However, a full comparison of these two techniques is beyond the scope of this study and has been reviewed elsewhere (Aibar et al., 2017; Chen, Ning, & Shi, 2019; Fiers et al., 2018; Lamere & Li, 2019). One caveat to WGCNA analysis is that once a gene is grouped into a gene regulatory network, which in the case of WGCNA is a group of co-expressed genes termed a module, it cannot be grouped with another module. This may not be entirely reflective of the potentially multi-functional nature of a given gene. In contrast, SCENIC allows genes to be grouped into more than one gene regulatory network, which in the case of SCENIC is a transcription factor and its downstream genes with confirmed binding site motifs termed a regulon. Despite this caveat, WGCNA, which groups genes by co-expression, may offer an advantage over SCENIC since it does not attempt to link a transcription factor directly with downstream genes, and may identify more indirect relationships between genes in a given module and may include both genes upstream and downstream from given genes.

**Quantitation of novel cell type-specific gene transcripts supports cell type-specificity of transcripts within the SV.** Customized MATLAB code was utilized to determine the expression of novel gene transcripts in SV cell type nuclei. The percentage of cell type-specific nuclei labeled with candidate cell type-specific smFISH probes was quantified (Supplemental Figure S5A). Fifty-two of 56 (93%) and 66 of 66 (100%) of marginal cell nuclei expressed *Abcg1* and *Heyl* transcripts, respectively. One hundred thirty seven of 161 (85%) and 170 of 176 (97%) of *Kcnj10*- and *Cd44*-expressing intermediate cell nuclei expressed *Nrp2* and *Kcnj13* transcripts, respectively. 107 of 145 (73%) and 118 of 185 (64%) of basal cell nuclei express *Sox8* (n=145 cells) and *Nr2f2* (n=185 cells) transcripts, respectively.

**smFISH demonstrates regulon transcription factor co-expression with downstream gene targets.** Of the Esrrb transcript-positive nuclei, 93% were *Abcg1*-positive (52 of 56 nuclei), 100% (59 of 59 nuclei) were *Atp13a5*-positive, and 100% (66 of 66 nuclei) were *Heyl*-positive (Supplemental Figure S5B). Of the Bmyc transcript-positive nuclei, 93% (40 of 43 nuclei) were *Cd44*-positive, 97% (36 of 37 nuclei) were *Met*-positive, and 87% (33 of 38 nuclei) were *Pax3*-positive (Supplemental Figure S5C). The high percentage of co-expression of the respective transcription factors and their downstream target genes suggests that these regulons exist in the P30 stria vascularis and provide support for the validity of the entire regulon.

**Review of novel SV genes**

*Abcg1* is a lipid transporter that appears to be involved in regulation of cholesterol homeostasis at the blood-brain barrier and mutations in Abcg1 have been implicated in impaired monocyte cholesterol clearance in age-related macular degeneration resulting in vision loss (Ban et al., 2018; Kober et al., 2017; Tarling, 2013). Prior to this study, *Abcg1* expression had not been demonstrated in the cochlea. Malgrange and colleagues have suggested the possibility that cholesterol homeostasis plays a role in the development of SNHL and that genes involved may offer the possibility of therapeutic approaches to treat or prevent SNHL (Malgrange, Varela-Nieto, de Medina, & Paillasse, 2015). *Heyl* is a downstream effector of the Notch signaling pathway and has been shown to be expressed in the developing organ of Corti, appearing to be responsible for maintaining the fate of cochlear supporting cells (Doetzlhofer et al., 2009; McGovern, Zhou, Randle, & Cox, 2018). However, prior to this study, *Heyl* expression had not been demonstrated in the adult cochlea, although presumably it plays a role maintaining cell fate. In addition to its role in axon guidance, *Nrp2* has been recently demonstrated to be expressed in the cuboidal cells of the avian tegmentum vasculosum, the avian equivalent to the mammalian SV (Coate, Spita, Zhang, Isgrig, & Kelley, 2015; Scott, Yue, Biesemeier, Lee, & Fekete, 2019). A conditional knockout mouse of an *Nrp2* paralog, *Nrp1*, has been associated with enlarged microvessels of the SV and *Nrp1* is expressed in the developing SV at postnatal day 5 (Salehi et al., 2017). *Kcnj13*, encodes a potassium inwardly-rectifying channel known as Kir7.1, has been identified as being expressed by adult cochlear hair cell and supporting cell transcriptome profiles but has not been demonstrated to be expressed in the adult SV (Liu et al., 2018, 2014). *Sox8* is a transcription factor involved in initial signaling and maintenance of *Sox10* expression in the otic placode during inner ear development (Betancur, Sauka-Spengler, & Bronner, 2011). While *Sox8* has been shown to be expressed in the developing chicken otocyst in the region of the tegmentum vasculosum (Sinkkonen et al., 2011), the equivalent to the mammalian SV, it has not been previously demonstrated in the mammalian stria vascularis, much less the adult SV. *Nr2f2* is an orphan steroid/thyroid hormone nuclear receptor with an essential role in angiogenesis during development and known expression in embryonic and adult mouse cochlear and vestibular sensory epithelia (Tornari, Towers, Gale, & Dawson, 2014). Expression of *Nr2f2* has not been previously demonstrated in the SV. *Kcnj16*, encodes a potassium inwardly-rectifyng channel known as Kir5.1, has been identified as being expressed by adult cochlear hair cell and supporting cell transcriptome profiles but has not been demonstrated to be expressed in the adult SV (Liu et al., 2018, 2014). *P2rx2*, encodes the P2X2 ATP-sensitive non-specific cation channel which is known to be expressed in outer sulcus cells but previously described as being absent in the SV (Järlebark, Housley, Raybould, Vlajkovic, & Thorne, 2002; Järlebark, Housley, & Thorne, 2000). We demonstrate *P2rx2* transcript expression in SV spindle cells (Figure 3E, 3F). Finally, *Atp13a5* is a P5 ATPase that has been demonstrated to be highly expressed in the adult mouse brain (Schultheis et al., 2004; Weingarten, Dave, Li, & Crawford, 2012). *Atp13a5* has been shown to be expressed in transcriptome profiles from whole mouse cochlea from early postnatal mice but has not been previously localized within the SV (Son et al., 2012).

**Supplemental Methods**

**Aggregation of multiple cell captures for single cell RNA-Seq of the adult mouse stria vascularis.** Three captures of single cells from P30 mouse stria vascularis were performed using 5 mice for the first cell capture and 10 mice per capture for the last 2 cell captures. Datasets from all 3 captures were aggregated in CellRanger. Distribution of cells across all clusters was analyzed and found to be equally distributed across all clusters based on cell capture date (Supplemental Figure S1). For these reasons, the dataset was treated as a single dataset. No other batch correction methods were used prior to analyzing these datasets. One capture of single nuclei from P30 mouse stria vascularis was performed using 10 mice.

**Quantification of dissociation-induced gene expression in scRNA-Seq and snRNA-Seq datasets from the adult mouse stria vascularis.** In order to compare the dissociation genes' expression between scRNASeq and snRNASeq data, we normalized the average expression level of the dissociation genes to three nuclear loading controls: *Lmnb1*, *Pcna*, *Tbp* (Akiyama et al., 2013; Iwai et al., 2019; Prior et al., 2014; Zhao et al., 2019). A composite normalization factor was created from these 3 nuclear gene controls, as both datasets had material from the nucleus. This normalization factor was utilized to create a mean estimate of total cellular content that would be less sensitive to the technical or biological fluctuations of a single loading control (Janes, 2015). Briefly, the following procedure was followed: (1) The average expression of *Lmnb1*, *Pcna*, and *Tbp* in each dataset was calculated as the normalization factor, respectively; (2) The expression of dissociation-induced genes in each cell was normalized to the normalization factor calculated from (1); (3) The average expression of all the dissociation genes in each cell was calculated; and (4) Results were plotted as a boxplot using Seaborn (v0.9). A paired 2-tailed Student’s t-test was used for the pairwise comparison of dissociation-induced gene expression between scRNA-Seq and snRNA-Seq datasets.

**Procedure for filtering dissociation gene artifacts.** After importation of raw RNA-Seq data into R, rows (genes) of the expression matrix were removed using a master list of known dissociation genes (Van Den Brink et al., 2017). Subsequently, doublets were filtered from the data based on the results from the DoubletDecon package described previously (DePasquale et al., 2018) and the standard Seurat pipeline was followed. The resultant clustering was then compared with that of the pre-dissociative gene filtered clustering.

**Projection of WGCNA modules and SCENIC regulons onto Seurat objects.** To project WGCNA modules onto Seurat objects, we imported the gene list of the WGCNA module of interest, converted them to a character vector, and added them to the “2.6_regulons_asGeneSet” file in the /int folder of the SCENIC file under a new name. This is the file that SCENIC recognizes as the master list of regulon names and gene lists. The “runSCENIC3” function, part of which is the regulon scoring for cells, is re-run so that the new pseudo-regulon of WGCNA module genes will be scored in addition to the SCENIC regulons. We then loaded the SCENIC expression files and Seurat object and substituted the TSNE coordinates of the SCENIC clustering with the TSNE coordinates of the Seurat object, and used the name of the new “WGCNA regulon” as the argument for projection.

To project SCENIC regulons onto Seurat objects, we loaded the SCENIC expression files and Seurat object and simply substituted the TSNE coordinates of the SCENIC clustering with the TSNE coordinates of the Seurat object. Supplemental code is provided (Supplemental File: Data Sheet 1.docx).

**Semi-supervised quantification of smFISH transcript expression for novel marker genes and regulon validation.** To quantify novel marker gene expression and regulon expression within a given cell type, we developed a semi-supervised image processing tool to quantify the number of smFISH signal dots, which represent RNA transcript expression, and quantified the average intensity of these signal dots in a given region of interest. The graphical user interface allows for the following regions of interest to be selected: cells or nuclei, 3 circles of a selected size, or a freehand outlined region. Maximum intensity projection images with and without DAPI signal are imported. Once all regions of interest (cells or nuclei, 3 circles, or a freehand outlined region) are selected, then the tool outputs a table with the following columns for counts (red, green, blue) and intensities (red, green, blue). In this study, nuclei were circled for the respective smFISH probe and were counted as positive if they had any quantified signal for a given smFISH probe. Customized MATLAB code is provided (Supplemental File: Data Sheet 2.docx).

The percentage of each cell type-specific nuclei (marginal, intermediate, and basal cells) that demonstrated transcript expression by smFISH of each novel cell type-specific gene were calculated. Where possible, co-expression with smFISH probes of known cell type-specific genes was performed in conjunction with candidate probes.

To validate the *Esrrb* and *Bmyc* regulons, we chose downstream targets with commercially-available RNAScope probes and proceeded to demonstrate their co-expression with the regulon transcription factor in marginal (*Esrrb*) and intermediate (*Bmyc*) nuclei, respectively. For each regulon, the number of nuclei with *Esrrb* or *Bmyc* transcripts was utilized as the reference number of cells for each regulon and the number of Esrrb-positive or Bmyc-positive nuclei with transcripts for each of the respective downstream target smFISH probes was determined. For each regulon transcription factor-downstream gene target pair, separate sections were utilized.

**Supplemental References**

Aibar, S., González-Blas, C. B., Moerman, T., Huynh-Thu, V. A., Imrichova, H., Hulselmans, G., … Aerts, S. (2017). SCENIC: Single-cell regulatory network inference and clustering. *Nature Methods*. https://doi.org/10.1038/nmeth.4463

Akiyama, M., Liew, C. W., Lu, S., Hu, J., Martinez, R., Hambro, B., … Kulkarni, R. N. (2013). X-Box binding protein 1 is essential for insulin regulation of pancreatic α-cell function. *Diabetes*. https://doi.org/10.2337/db12-1747

Ando, M., & Takeuchi, S. (1999). Immunological identification of an inward rectifier K+ channel (Kir4.1) in the intermediate cell (melanocyte) of the cochlear stria vascularis of gerbils and rats. *Cell and Tissue Research*. https://doi.org/10.1007/s004419900066

Bakken, T. E., Hodge, R. D., Miller, J. A., Yao, Z., Nguyen, T. N., Aevermann, B., … Tasic, B. (2018). Single-nucleus and single-cell transcriptomes compared in matched cortical cell types. *PLoS ONE*. https://doi.org/10.1371/journal.pone.0209648

Ban, N., Lee, T. J., Sene, A., Choudhary, M., Lekwuwa, M., Dong, Z., … Apte, R. S. (2018). Impaired monocyte cholesterol clearance initiates age-related retinal degeneration and vision loss. *JCI Insight*. https://doi.org/10.1172/jci.insight.120824

Baryawno, N., Przybylski, D., Kowalczyk, M. S., Kfoury, Y., Severe, N., Gustafsson, K., … Scadden, D. T. (2019). A Cellular Taxonomy of the Bone Marrow Stroma in Homeostasis and Leukemia. *Cell*. https://doi.org/10.1016/j.cell.2019.04.040

Betancur, P., Sauka-Spengler, T., & Bronner, M. (2011). A sox10 enhancer element common to the otic placode and neural crest is activated by tissue-specific paralogs. *Development*. https://doi.org/10.1242/dev.057836

Cable, J., & Steel, K. P. (1991). Identification of Two Types of Melanocyte Within the Stria Vascularis of the Mouse Inner Ear. *Pigment Cell Research*. https://doi.org/10.1111/j.1600-0749.1991.tb00320.x

Chen, G., Ning, B., & Shi, T. (2019). Single-cell RNA-seq technologies and related computational data analysis. *Frontiers in Genetics*. https://doi.org/10.3389/fgene.2019.00317

Coate, T. M., Spita, N. A., Zhang, K. D., Isgrig, K. T., & Kelley, M. W. (2015). Neuropilin-2/semaphorin-3F-mediated repulsion promotes inner hair cell innervation by spiral ganglion neurons. *ELife*. https://doi.org/10.7554/eLife.07830

DePasquale, E. A. K., Schnell, D. J., Valiente, I., Blaxall, B. C., Grimes, H. L., Singh, H., & Salomonis, N. (2018). DoubletDecon: Cell-State Aware Removal of Single-Cell RNA-Seq Doublets. *BioRxiv*, 364810. https://doi.org/10.1101/364810

Doetzlhofer, A., Basch, M. L., Ohyama, T., Gessler, M., Groves, A. K., & Segil, N. (2009). Hey2 Regulation by FGF Provides a Notch-Independent Mechanism for Maintaining Pillar Cell Fate in the Organ of Corti. *Developmental Cell*. https://doi.org/10.1016/j.devcel.2008.11.008

Fiers, M. W. E. J., Minnoye, L., Aibar, S., González-Blas, C. B., Atak, Z. K., & Aerts, S. (2018). Mapping gene regulatory networks from single-cell omics data. *Briefings in Functional Genomics*. https://doi.org/10.1093/bfgp/elx046

Habib, N., Li, Y., Heidenreich, M., Swiech, L., Avraham-Davidi, I., Trombetta, J. J., … Regev, A. (2016). Div-Seq: Single-nucleus RNA-Seq reveals dynamics of rare adult newborn neurons. *Science*. https://doi.org/10.1126/science.aad7038

Hu, P., Liu, J., Zhao, J., Wilkins, B. J., Lupino, K., Wu, H., & Pei, L. (2018). Single-nucleus transcriptomic survey of cell diversity and functional maturation in postnatal mammalian hearts. *Genes and Development*. https://doi.org/10.1101/gad.316802.118

Iwai, K., Nambu, T., Dairiki, R., Ohori, M., Yu, J., Burke, K., … Ohashi, A. (2019). Molecular mechanism and potential target indication of TAK-931, a novel CDC7-selective inhibitor. *Science Advances*. https://doi.org/10.1126/sciadv.aav3660

Janes, K. A. (2015). An analysis of critical factors for quantitative immunoblotting. *Science Signaling*. https://doi.org/10.1126/scisignal.2005966

Järlebark, L. E., Housley, G. D., Raybould, N. P., Vlajkovic, S., & Thorne, P. R. (2002). ATP-gated ion channels assembled from P2X2receptor subunits in the mouse cochlea. *NeuroReport*.

Järlebark, L. E., Housley, G. D., & Thorne, P. R. (2000). Immunohistochemical localization of adenosine 5’-triphosphate-gated ion I channel P2X2receptor subunits in adult and developing rat cochlea. *Journal of Comparative Neurology*. https://doi.org/10.1002/(SICI)1096-9861(20000605)421:3<289::AID-CNE1>3.0.CO;2-0

Kober, A. C., Manavalan, A. P. C., Tam-Amersdorfer, C., Holmér, A., Saeed, A., Fanaee-Danesh, E., … Panzenboeck, U. (2017). Implications of cerebrovascular ATP-binding cassette transporter G1 (ABCG1) and apolipoprotein M in cholesterol transport at the blood-brain barrier. *Biochimica et Biophysica Acta - Molecular and Cell Biology of Lipids*. https://doi.org/10.1016/j.bbalip.2017.03.003

Lacar, B., Linker, S. B., Jaeger, B. N., Krishnaswami, S., Barron, J., Kelder, M., … Gage, F. H. (2016). Nuclear RNA-seq of single neurons reveals molecular signatures of activation. *Nature Communications*. https://doi.org/10.1038/ncomms11022

Lake, B. B., Ai, R., Kaeser, G. E., Salathia, N. S., Yung, Y. C., Liu, R., … Zhang, K. (2016). Neuronal subtypes and diversity revealed by single-nucleus RNA sequencing of the human brain. *Science*. https://doi.org/10.1126/science.aaf1204

Lamere, A. T., & Li, J. (2019). Inference of gene co-expression networks from single-cell RNA-sequencing data. In *Methods in Molecular Biology*. https://doi.org/10.1007/978-1-4939-9057-3_10

Langfelder, P., & Horvath, S. (2008). WGCNA: An R package for weighted correlation network analysis. *BMC Bioinformatics*. https://doi.org/10.1186/1471-2105-9-559

Liu, H., Chen, L., Giffen, K. P., Stringham, S. T., Li, Y., Judge, P. D., … He, D. Z. Z. (2018). Cell-Specific Transcriptome Analysis Shows That Adult Pillar and Deiters’ Cells Express Genes Encoding Machinery for Specializations of Cochlear Hair Cells. *Frontiers in Molecular Neuroscience*. https://doi.org/10.3389/fnmol.2018.00356

Liu, H., Pecka, J. L., Zhang, Q., Soukup, G. A., Beisel, K. W., & He, D. Z. Z. (2014). Characterization of transcriptomes of cochlear inner and outer hair cells. *Journal of Neuroscience*. https://doi.org/10.1523/JNEUROSCI.1690-14.2014

Malgrange, B., Varela-Nieto, I., de Medina, P., & Paillasse, M. R. (2015). Targeting cholesterol homeostasis to fight hearing loss: A new perspective. *Frontiers in Aging Neuroscience*. https://doi.org/10.3389/fnagi.2015.00003

McGovern, M. M., Zhou, L., Randle, M. R., & Cox, B. C. (2018). Spontaneous Hair Cell Regeneration Is Prevented by Increased Notch Signaling in Supporting Cells. *Frontiers in Cellular Neuroscience*. https://doi.org/10.3389/fncel.2018.00120

Prior, S., Kim, A., Yoshihara, T., Tobita, S., Takeuchi, T., & Higuchi, M. (2014). Mitochondrial respiratory function induces endogenous hypoxia. *PLoS ONE*. https://doi.org/10.1371/journal.pone.0088911

Salehi, P., Ge, M. X., Gundimeda, U., Michelle Baum, L., Lael Cantu, H., Lavinsky, J., … Friedman, R. A. (2017). Role of Neuropilin-1/Semaphorin-3A signaling in the functional and morphological integrity of the cochlea. *PLoS Genetics*. https://doi.org/10.1371/journal.pgen.1007048

Sathyamurthy, A., Johnson, K. R., Matson, K. J. E., Dobrott, C. I., Li, L., Ryba, A. R., … Levine, A. J. (2018). Massively Parallel Single Nucleus Transcriptional Profiling Defines Spinal Cord Neurons and Their Activity during Behavior. *Cell Reports*. https://doi.org/10.1016/j.celrep.2018.02.003

Schultheis, P. J., Hagen, T. T., O’Toole, K. K., Tachibana, A., Burke, C. R., McGill, D. L., … Shull, G. E. (2004). Characterization of the P 5 subfamily of P-type transport ATPases in mice. *Biochemical and Biophysical Research Communications*. https://doi.org/10.1016/j.bbrc.2004.08.156

Scott, M. K., Yue, J., Biesemeier, D. J., Lee, J. W., & Fekete, D. M. (2019). Expression of class III Semaphorins and their receptors in the developing chicken (Gallus gallus) inner ear. *Journal of Comparative Neurology*. https://doi.org/10.1002/cne.24595

Sinkkonen, S. T., Starlinger, V., Galaiya, D. J., Laske, R. D., Myllykangas, S., Oshima, K., & Heller, S. (2011). Serial analysis of gene expression in the chicken otocyst. *JARO - Journal of the Association for Research in Otolaryngology*. https://doi.org/10.1007/s10162-011-0286-z

Son, E. J., Wu, L., Yoon, H., Kim, S., Choi, J. Y., & Bok, J. (2012). Developmental gene expression profiling along the tonotopic axis of the mouse cochlea. *PLoS ONE*. https://doi.org/10.1371/journal.pone.0040735

Tarling, E. (2013). Expanding roles of ABCG1 and sterol transport. *Current Opinion in Lipidology*. https://doi.org/10.1097/MOL.0b013e32835da122

Tornari, C., Towers, E. R., Gale, J. E., & Dawson, S. J. (2014). Regulation of the orphan nuclear receptor Nr2f2 by the DFNA15 deafness gene Pou4f3. *PLoS ONE*. https://doi.org/10.1371/journal.pone.0112247

Van Den Brink, S. C., Sage, F., Vértesy, Á., Spanjaard, B., Peterson-Maduro, J., Baron, C. S., … Van Oudenaarden, A. (2017). Single-cell sequencing reveals dissociation-induced gene expression in tissue subpopulations. *Nature Methods*. https://doi.org/10.1038/nmeth.4437

Wangemann, P., Itza, E. M., Albrecht, B., Wu, T., Jabba, S. V., Maganti, R. J., … Marcus, D. C. (2004). Loss of KCNJ10 protein expression abolishes endocochlear potential and causes deafness in Pendred syndrome mouse model. *BMC Medicine*. https://doi.org/10.1186/1741-7015-2-30

Weingarten, L. S., Dave, H., Li, H., & Crawford, D. A. (2012). Developmental expression of P5 ATPase mRNA in the mouse. *Cellular and Molecular Biology Letters*. https://doi.org/10.2478/s11658-011-0039-3

Wu, H., Kirita, Y., Donnelly, E. L., & Humphreys, B. D. (2019a). Advantages of single-nucleus over single-cell RNA sequencing of adult kidney: Rare cell types and novel cell states revealed in fibrosis. *Journal of the American Society of Nephrology*. https://doi.org/10.1681/ASN.2018090912

Wu, H., Kirita, Y., Donnelly, E. L., & Humphreys, B. D. (2019b). Advantages of Single-Nucleus over Single-Cell RNA Sequencing of Adult Kidney: Rare Cell Types and Novel Cell States Revealed in Fibrosis. *Journal of the American Society of Nephrology*. https://doi.org/10.1681/asn.2018090912

Yang, S., Corbett, S. E., Koga, Y., Wang, Z., Johnson, W. E., Yajima, M., & Campbell, J. D. (2019). Decontamination of ambient RNA in single-cell RNA-seq with DecontX. *BioRxiv*. https://doi.org/10.1101/704015

Zeng, W., Jiang, S., Kong, X., El-Ali, N., Ball, A. R., Ma, C. I. H., … Mortazavi, A. (2016). Single-nucleus RNA-seq of differentiating human myoblasts reveals the extent of fate heterogeneity. *Nucleic Acids Research*. https://doi.org/10.1093/nar/gkw739

Zhao, K., Jia, Y., Peng, J., Pang, C., Zhang, T., Han, W., … Qian, Y. (2019). Anacardic acid inhibits RANKL-induced osteoclastogenesis in vitro and prevents ovariectomy-induced bone loss in vivo . *The FASEB Journal*. https://doi.org/10.1096/fj.201802575rr
